# Integrated bioassays and metabolomics identify *Cleome noeana* as a promising antibacterial candidate

**DOI:** 10.3389/fmicb.2026.1777863

**Published:** 2026-03-27

**Authors:** Ali El-Keblawy, Ohood Sallam, Tareq M. Osaili, Fouad Lamghari, Aseela Al Moalla, Mohamed Sheteiwy, Khaloud Mohammed Alarjani, Soumya Koippully Manikandan, Attiat Elnaggar

**Affiliations:** 1 Department of Applied Biology, College of Sciences, University of Sharjah, Sharjah, United Arab Emirates; 2 Faculty of Pharmacy, Al Salam University, Tanta, Egypt; 3 Faculty of Science, Botany and Microbiology Department, Menoufia University, Shebeen El-Kom, Egypt; 4 Research Institute for Medical and Health Sciences, University of Sharjah, Sharjah, United Arab Emirates; 5 Department of Clinical Nutrition and Dietetics, College of Health Sciences, University of Sharjah, Sharjah, United Arab Emirates; 6 Department of Nutrition and Food Technology, Faculty of Agriculture, Jordan University of Science and Technology, Irbid, Jordan; 7 Fujairah Research Centre, Fujairah, United Arab Emirates; 8 Fujairah Environment Authority, Sakamkam - Al Hilal City, Fujairah, United Arab Emirates; 9 Department of Integrative Agriculture, College of Agriculture and Veterinary Medicine, United Arab Emirates University, Al Ain, United Arab Emirates; 10 Department of Botany and Microbiology, College of Science, King Saud University, Riyadh, Saudi Arabia; 11 Advanced Biotechnology Research Center, Research Institute of Sciences and Engineering, University of Sharjah, Sharjah, United Arab Emirates; 12 Department of Botany and Microbiology, College of Science, Alexandria University, Alexandria, Egypt

**Keywords:** 25-hydroxycholesterol, antimicrobial resistance, *Cleome noeana* (syn. C. quinquenervia), desert medicinal plants, GC–MS metabolomics, phage–plant interaction, time–kill kinetics

## Abstract

Antimicrobial resistance has intensified the search for novel antibacterial agents from underexplored desert flora. This study compared three arid-region *Cleome* species to identify the most active candidate against *Escherichia coli, Salmonella enterica*, and *Staphylococcus aureus*, and to relate its antibacterial performance to metabolite composition and compatibility with bacteriophage treatment. An *in vitro* workflow combined crude-extract antibacterial screening, agar diffusion assays, time-kill kinetics, and untargeted GC–MS metabolomics coupled with multivariate analysis. Among the tested species, *Cleome noeana* exhibited the broadest and strongest antibacterial activity, with minimum inhibitory concentrations of 6.25–25 mg/mL and MBC/MIC ratios ≤ 4. Inhibition zone diameters were inversely correlated with MIC values. Time-kill assays demonstrated that *C. noeana* extracts reduced viable bacterial counts more rapidly than bacteriophages or antibiotics alone, while extract–phage combinations achieved >99% inhibition within 4 h, indicating phage compatibility with accelerated killing rather than true synergism. Untargeted metabolomic profiling tentatively associated antibacterial activity with phenolic acids, terpenoids, and long-chain fatty acids, including caffeic acid, α-terpineol, and myristic acid. Overall, *Cleome noeana* emerges as a promising antibacterial species from desert ecosystems and a plant extract compatible with bacteriophage-based approaches, supporting further targeted isolation and *in vivo* evaluation of its bioactive constituents.

## 1 Introduction

Antimicrobial resistance (AMR) is a mounting global health threat, now recognized as one of the leading causes of death worldwide. In 2019 alone, an estimated 1.27 million deaths were directly attributable to bacterial AMR, and 4.95 million were associated with drug-resistant infections, a toll that surpasses those of HIV/AIDS and malaria (Murray et al., 2022). The World Health Organization's 2024 Bacterial Priority Pathogens List further emphasizes the urgent need for new therapies targeting *Escherichia coli, Salmonella*, and *Staphylococcus aureus*, all of which are now associated with rising resistance and limited treatment options (WHO, 2024).

Against this backdrop, natural products continue to serve as a foundational source of antibiotic innovation. Over two-thirds of antibiotics in clinical use are derived from or inspired by natural metabolites. Plants in particular are rich in phenolics, terpenoids, alkaloids, and fatty acids, which exhibit antimicrobial effects through mechanisms distinct from conventional antibiotics, including membrane disruption, efflux pump inhibition, oxidative stress induction, and antibiofilm activity (Porras et al., 2020). Plants adapted to deserts and other extreme environments represent promising sources of novel bioactive compounds. Chronic exposure to heat, UV radiation, drought, and oxidative stress drives the evolution of chemically unique secondary metabolites, many of which remain poorly characterized. Such ecosystems function as natural laboratories, where selective pressures favor compounds with ecological roles in defense, allelopathy, and microbial competition, making desert plants an underexplored reservoir of antimicrobial leads (Ofir, 2020). Recent studies illustrate this adaptive chemistry across arid flora. For example, *Saposhnikovia divaricata*, a medicinal herb from arid grasslands, increases glycosides and aglycones under drought stress (Cao et al., 2022), while *Nitraria sibirica* accumulates flavonoids through abscisic-acid–regulated pathways that enhance antioxidant defenses during drought conditions (Chang et al., 2024). Metabolomic profiling of *Haloxylon ammodendron* and *H. persicum* has further revealed lignans, neolignans, organic acids, and alkaloid derivatives associated with osmotic balance, reactive oxygen species detoxification, and membrane stabilization (Yang and Lv, 2023). Comparative work further shows convergent strategies across desert flora: Atacama Desert species enrich drought- and nitrogen-stress pathways (Dussarrat et al., 2024), while the extremophyte *Anastatica hierochuntica* displays heat-induced transcriptomic flexibility and UV-B repair gene selection (Eshel et al., 2022). Together, these findings reinforce that desert plants and their microbiomes are untapped sources of antimicrobial and stress-protective metabolites, evolved under selective pressures rarely found in other ecosystems.

Several species within the *Cleome* genus already demonstrate strong antibacterial activity. For example, *C. spinosa* leaf extracts inhibit *Staphylococcus aureus* with minimum inhibitory concentrations (MICs) below 1 mg/mL and show synergistic effects when combined with oxacillin (Silva et al., 2016). Similarly, essential oils from *C. droserifolia* have been found to suppress the growth of *S. aureus, Escherichia coli*, and *Salmonella* species (Muhaidat et al., 2015). In addition, the ethanolic extract of *C. arabica* has demonstrated antibacterial activity against both Gram-positive and Gram-negative bacteria (Alkhatib et al., 2022). Despite these findings, **desert-endemic** ***Cleome* species, including** *C. amblyocarpa, C. rupicola*, **and** *C. noeana***, remain largely understudied**. While *C. amblyocarpa* and *C. rupicola* have been the subject of only preliminary compositional or antioxidant investigations, **no indexed studies to date have evaluated the antibacterial potential of** *Cleome noeana*, a taxon sometimes synonymized with *C. fimbriata* and commonly distributed in arid habitats in the Middle East. This lack of data highlights a significant gap in current research.

To address this knowledge gap, we evaluated the antibacterial activity of three underexplored desert species, *C. amblyocarpa, C. rupicola*, and *C. noeana*, using a combination of *in vitro* bioassays (agar diffusion, MIC/MBC thresholds), untargeted GC–MS metabolomics, and chemometric modeling to identify candidate bioactives. We further tested *C. noeana* the extract in combination with lytic bacteriophages, given the growing interest in phage–antibiotic synergy as a strategy to combat resistance. While synergy between phages and antibiotics is well documented (Gu Liu et al., 2020; Qin et al., 2024), synergy between phages and plant extracts remains rare and unpredictable, ranging from potent synergy (e.g., *Alhagi maurorum* extract + phage: 73% biofilm reduction) to clear antagonism (e.g., *Echinacea* extract inhibiting phage infectivity) (Mirzaei et al., 2024; Stachurska et al., 2023).

We hypothesized that *C. noeana*, as a desert-adapted species, would harbor unique secondary metabolites with broad-spectrum antibacterial activity, and that these compounds might exhibit additive or synergistic effects with phage therapy. By integrating bioassay-guided screening with metabolomic profiling and phage combination testing, this study aims to identify a new antibacterial lead from arid flora and demonstrate its potential in dual-agent antimicrobial applications.

## 2 Materials and methods

### 2.1 Plant sampling and extraction

Leaf samples of three arid-region *Cleome* species: *C. amblyocarpa, C. rupicola*, and *C. noeana* were collected from the Fujairah and Sharjah emirates, United Arab Emirates, during May 2024. Representative voucher specimens from Sharjah collection sites are reported here, while additional sampling was conducted across both the Sharjah and Fujairah emirates.

#### 2.1.1 Botanical authentication and voucher

The taxa were identified by Prof. Ali El-Keblawy (Department of Applied Biology, University of Sharjah) using regional floras and verified morphological characteristics. Voucher specimens were deposited in the University of Sharjah Herbarium (UOS), Sharjah, UAE, under the following accession numbers: *Cleome noeana* Boiss. (UOS-CNO-2024-05-507; Sharjah – Khor Fakkan Road, 25°12′59″ N 56°00′51″ E); *C. rupicola* Benth. (UOS-CRP-2024-05-508; Sharjah – Kalba Road, 25°04′47″ N 56°02′05″ E); and *C. amblyocarpa* Boiss. (UOS-CMB-2024-05-509; Al Ramaqiya, Sharjah, 25°09′42″ N 55°38′35″ E). All plant materials were collected within publicly accessible areas of the Emirate of Sharjah where no special collection permit was required.

#### 2.1.2 Extraction procedure

Collected leaves were washed with tap water, rinsed with distilled water to remove surface impurities, and air-dried at ambient temperature in darkness. The dried material was ground into a fine powder using a Tissue Lyser II (Model 1218200424E, Qiagen, Hilden, Germany). Extraction was carried out in 70 % ethanol at a ratio of 1:6 (w/v; 1 g powder per 6 mL solvent). Samples were incubated for 24 h on a rotary shaker at room temperature and then sonicated in a water bath for 1 h. Filtrates were obtained through Whatman No. 1 filter paper, and the residues were re-extracted three times with fresh solvent to maximize metabolite recovery. Combined filtrates were concentrated under reduced pressure using a rotary evaporator (Rotavapor^®^ R-300, Büchi, Switzerland), frozen at −80 °C, and freeze-dried in a lyophilizer (Model 700201050, Labconco, USA). The resulting lyophilized extracts were stored at 4 °C until further analysis (Hamdy et al., 2025).

### 2.2 Gas chromatography–mass spectrometry (GC–MS) analysis

Fifty microliters of hexane and 20 μL of methoxyamine hydrochloride (20 mg/mL in pyridine) were added to the dried leaf extracts. After vortexing, the samples were incubated at 37 °C for 1.5 h to allow for methoximation. Following this, 90 μL of N-methyl-N-(trimethylsilyl) trifluoroacetamide (MSTFA) containing 1% trimethylchlorosilane (TMCS) was added, and the mixture was further incubated for 1 h at 37 °C (Cohen et al., 2016; Moros et al., 2017).

GC–MS analysis was performed using a Shimadzu GC-2010 gas chromatograph coupled with a GCMS-QP2010 Ultra mass spectrometer, equipped with an AOC-20i + 20s autosampler (Shimadzu, Tokyo, Japan). Data acquisition and analysis were conducted using the Shimadzu MSD Enhanced ChemStation software.

Metabolite identification was conducted by comparing the mass fragmentation spectra to entries in the NIST 14 Mass Spectral Library. All compound identifications are considered putative unless verified using authentic reference standards. Special attention was paid to sterols and oxysterols, where isomeric overlap and derivatization artifacts may result in ambiguous spectral matches.

### 2.3 Antimicrobial and bacteriophage assays

#### 2.3.1 Bacterial strains and bacteriophage preparation

The antibacterial activity was evaluated against a panel of Gram-negative and Gram-positive reference bacterial strains. Gram-negative bacteria included *Escherichia coli* O157:H7 strain 161-84 (Canadian Science Center for Human and Animal Health, Ottawa, Canada), *Salmonella enterica* serovar Typhimurium strain 02-8423 (Health Canada, Ottawa), *Klebsiella pneumoniae* ATCC BAA-2146, and *Acinetobacter baumannii* ATCC 19606. Gram-positive bacteria comprised *Staphylococcus aureus* ATCC 29213, *Staphylococcus epidermidis* ATCC 14990, and *Streptococcus pneumoniae* ATCC 6301. All bacterial cultures were maintained on nutrient agar slants at 4 °C and sub-cultured in nutrient broth prior to testing.

Two strictly lytic bacteriophages were used in synergy assays: CF01, isolated from camel feces in Ajman, UAE, using an *E. coli* host, and SW01, isolated from municipal sewage in Ajman, UAE, using a *Salmonella enterica* host. CF01 exhibited a broader host range, lysing multiple *Escherichia coli* strains and several *Salmonella* isolates, whereas SW01 was specific to *Salmonella* but displayed a markedly higher burst size and productivity. Phages were propagated using the double-layer agar method, purified by centrifugation and filtration (0.22 μm), and stored at 4 °C until use (Kutter and Sulakvelidze, 2004). The bacteriophages used in this study were not re-characterized here, as their biological and genomic properties had been characterized previously by the authors. In the present work, phages were employed solely as well-defined biological agents to evaluate their synergistic antibacterial effects in combination with plant extracts.

#### 2.3.2 Antibacterial assays

##### 2.3.2.1 Disc diffusion assay

Antibacterial activity of the extracts was assessed using the disc diffusion method (Bauer et al., 1966). Sterile paper discs (6 mm) were impregnated with 30 μL of plant extract (100 mg/mL stock solution) and dried under sterile conditions before placement on Mueller–Hinton agar plates previously inoculated with standardized bacterial suspensions (0.5 McFarland standard, ~1.5 × 10^8^ CFU/mL). Plates were incubated at 37 °C for 18–24 h, and zones of inhibition were recorded in millimeters (Kaya et al., 2008; Dilbato Dinbiso et al., 2022).

##### 2.3.2.2 Well diffusion assay

The agar well diffusion assay was performed as a complementary qualitative test of antimicrobial activity (Balouiri et al., 2016). Wells (0.6 cm diameter) were bored into Mueller–Hinton agar seeded with bacterial inoculum, and 30 μL of plant extract was added to each well. Plates were left briefly at room temperature to allow diffusion, then incubated at 37 °C for 18–24 h, after which inhibition zones were measured (Janesha et al., 2020).

##### 2.3.2.3 Antibiotic controls

Chloramphenicol and tetracycline were used as positive control antibiotics in the disc diffusion and well diffusion assays. Commercial antibiotic discs containing chloramphenicol (30 μg/disc) and tetracycline (30 μg/disc) were employed in disc diffusion assays. For well diffusion assays, the same antibiotics were applied at a concentration of 100 μg/mL (30 μL per well), following standard antimicrobial susceptibility testing practices.

##### 2.3.2.4 Minimum inhibitory concentration (MIC) and minimum bactericidal concentration (MBC)

MIC values were determined using the broth microdilution method according to CLSI guidelines (CLSI, 2012). Stock solutions of each plant extract were prepared at 200 mg/mL in sterile solvent. Two-fold serial dilutions were made in Mueller–Hinton broth to obtain final concentrations ranging from 100 to 1.56 mg/mL. Each well received an equal volume of bacterial inoculum adjusted to a 0.5 McFarland standard. After incubation at 37 °C for 18–24 h, MIC was defined as the lowest concentration of extract that resulted in no visible bacterial growth. The minimum bactericidal concentration (MBC) was defined as the lowest concentration of extract that resulted in no detectable bacterial growth following sub-culturing onto agar plates, indicating complete bacterial killing (Andrews, 2001; CLSI, 2012). Antibiotic controls were not included in the MIC and MBC assays, as these experiments were designed specifically to determine the inhibitory and bactericidal concentrations of the plant extracts. Conventional antibiotics were used only as positive reference controls in the disc and well diffusion assays.

##### 2.3.2.5 Synergistic effect assay (phage + *Cleome noeana*)

The synergistic activity between *C. noeana* ethanolic extract and bacteriophages was evaluated against *E. coli* and *S. enterica*. Bacterial cultures were prepared at 1 × 10^6^ CFU/ml. **All experimental treatments were administered at 0 h, 4 h, and 24 h post-inoculation**, and included the following conditions:

1. Phage alone at MOI = 1 (phage concentration approximately equal to the bacterial concentration).
2. *C. noeana* extract alone at its MIC (determined as above).
3. Mixture of phage (MOI = 1) and *C. noeana* extract (at MIC).

Negative controls (bacterial cultures in nutrient broth without treatment) were included. All cultures were incubated at 37 °C. Viable bacterial counts (CFU/mL) were enumerated at each time point and compared to initial counts at 0 h to assess bactericidal effects. The design and endpoints followed established approaches for phage–antibiotic/antibacterial synergy studies and time-kill analyses (Nikolić et al., 2022; Kunz Coyne et al., 2024).

### 2.4 Statistical analysis

#### 2.4.1 Antimicrobial assays

Inhibition zone data from disc and well diffusion assays were averaged across replicates, and microbial isolates showing zero activity across all three *Cleome* species or no variance across species were excluded to avoid singularity. Two-way analysis of variance (ANOVA) was then performed separately for disc and well diffusion assays, with Plant species and Bacterial isolate as fixed factors (Zar, 2010). Because replicate MIC values within each Plant × Bacterium combination were identical (i.e., no within-cell variance) and some combinations were inactive within the tested range, classical ANOVA was not suitable for MIC. Instead, MICs were compared within each bacterium across plant species using Kruskal–Wallis tests (Kruskal and Wallis, 1952), followed by pairwise Mann–Whitney U tests (Mann and Whitney, 1947) with Holm correction for multiple comparisons (Holm, 1979). Descriptive statistics (median [IQR]) were reported in the main text, and full pairwise results are provided in the Supplementary material.

MIC and MBC values were calculated as means from three biological replicates. To classify bactericidal activity, the MBC/MIC ratio was determined, with values ≤ 4 interpreted as bactericidal according to accepted microbiological criteria. Relationships among inhibition zones, MIC, and MBC values were evaluated using Spearman's rank correlation coefficients, and results were visualized in correlation plots and heatmaps (Zar, 2010).

#### 2.4.2 Metabolite–activity integration

To integrate antimicrobial activity with metabolite profiles, GC–MS abundance data were aligned by species to form a species–metabolite matrix. Activity indices (mean inhibition zone [AvgZone], mean MIC, and mean MBC) were used as response variables. Pairwise associations between metabolite concentrations and activity indices were assessed using both Spearman and Pearson correlation coefficients to capture monotonic and linear trends, respectively.

For multivariate modeling, Partial Least Squares (PLS) regression was applied, with metabolite abundances as predictors (X) and antimicrobial activity indices as responses (Y). All variables were mean-centered and unit-variance scaled prior to analysis. Two latent components were retained to balance variance explanation and overfitting risk, given the small number of species. Variable Importance in Projection (VIP) scores were calculated following the approach of Chong and Jun (2005), with metabolites exceeding the threshold of VIP > 1 considered influential contributors to the activity model. Heatmaps were generated to visualize metabolite–activity correlations.

All analyses were conducted in Python (v3.11) using statsmodels v0.14.0 (ANOVA), SciPy v1.13.0 (Kruskal–Wallis, Mann–Whitney, correlations), scikit-learn v1.4.0 (PLS regression, VIP), Pandas v2.2.0 (data processing), and Matplotlib/Seaborn (visualization). A significance threshold of *p* < 0.05 was applied throughout.

## 3 Results

### 3.1 Antimicrobial activity of plant extracts

#### 3.1.1 Disc and well diffusion assays

Two-way ANOVA showed that *plant species, bacterial isolate*, and their interaction all significantly influenced inhibition zones in both disc and well diffusion assays (Table 1). The strong interaction terms indicate that the antibacterial efficacy of the extracts varied across bacterial species tested, rather than following a single uniform ranking.

**Table 1:** Two-way ANOVA of inhibition zones (disc and well diffusion assays) as affected by plant species, bacterial isolate, and their interaction.

Source	Type III SS	*df*	Mean squares	*F*-ratio	*p*-value
Disc diffusion					
Bacterial species	5.981	4	1.495	747.667	< 0.001
Plant species	13.636	2	6.818	3,409.000	< 0.001
Bacterial species × plant species	4.411	8	0.551	275.667	< 0.001
Error	0.060	30	0.002		
Well diffusion					
Bacterial species	9.619	4	2.405	1,082.150	< 0.001
Plant species	12.819	2	6.410	2,884.300	< 0.001
Bacterial species × plant species	2.354	8	0.294	132.425	< 0.001
Error	0.067	30	0.002		

**Figure 1:**
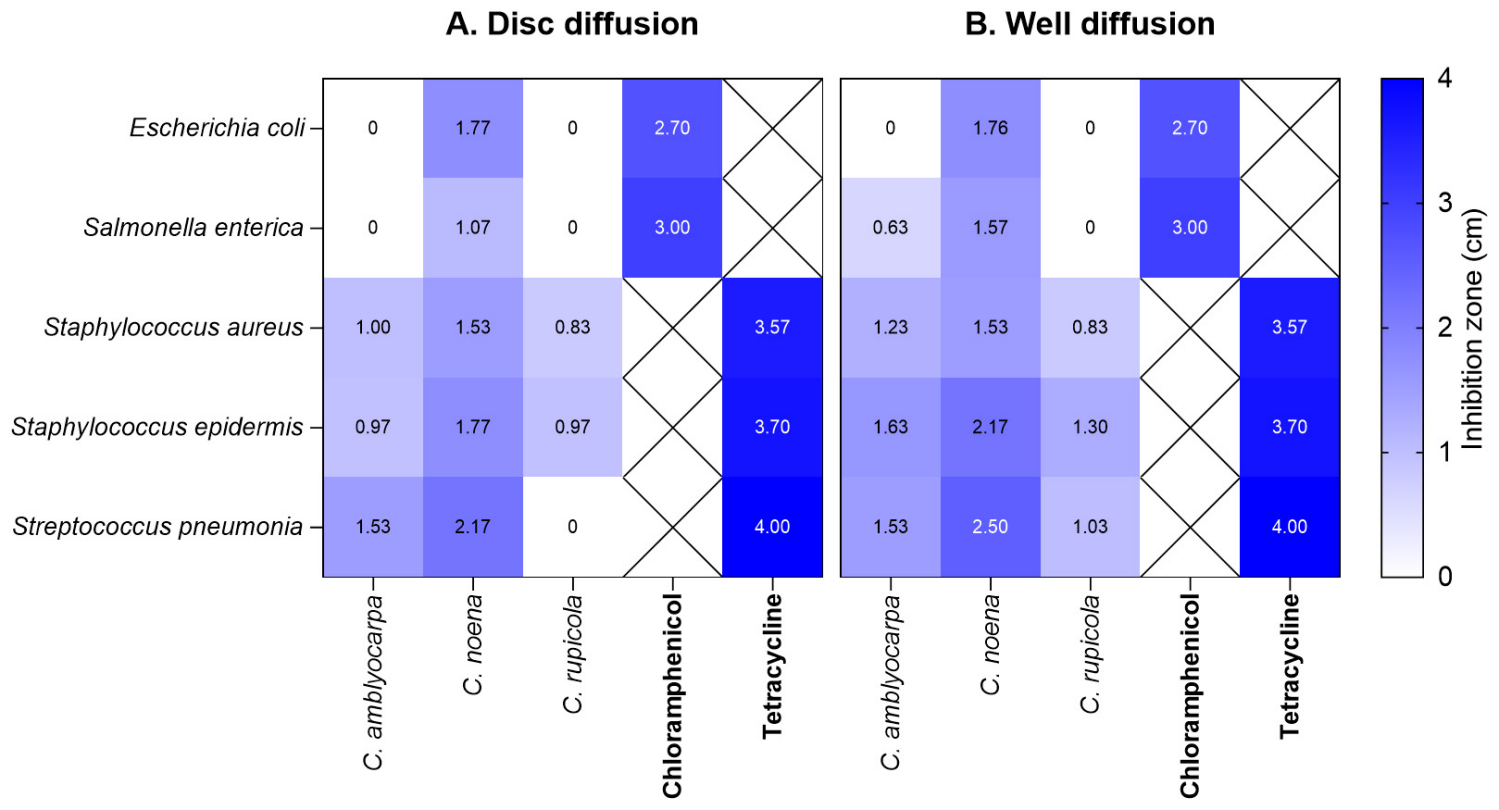
Antimicrobial activity of *Cleome* extracts in diffusion assays. **(A)** Disc diffusion and **(B)** Well diffusion heatmaps showing mean inhibition zones (cm) (based on three replicates) of the three plant species (*C. amblyocarpa, C. noeana*, and *C. rupicola)* and two positive controls (Chloramphenicol and Tetracycline) against bacterial isolates. Cells marked with X in the heatmap indicate no activity at the tested concentration.

The heatmaps (Figure 1) illustrate these trends clearly. The ethanolic extract of *Cleome noeana* consistently produced the largest inhibition zones, ranging from ~1.0 to 2.5 cm across isolates, and generally outperformed both *C. amblyocarpa* and *C. rupicola*. For example, against *Staphylococcus aureus* in disc diffusion, *C. noeana* extract achieved a mean zone of 1.6 cm compared with 1.0 cm for *C. amblyocarpa* (+60%) and 0.8 cm for *C. rupicola* (+100%). In well diffusion, *C. noeana* extract inhibited *Streptococcus pneumoniae* with a 2.5 cm zone, exceeding *C. amblyocarpa* (1.5 cm; +67%) and *C. rupicola* (1.0 cm; +150%).

Certain isolates, notably *Klebsiella pneumoniae* and *Acinetobacter baumannii*, showed no measurable inhibition from any of the three extracts in either assay, underscoring their relative resistance. In contrast, the positive controls (Chloramphenicol and Tetracycline) produced inhibition zones of 2.7–4.0 cm, representing ~1.2- to 1.8-fold greater activity than the strongest plant extracts.

#### 3.1.2 Minimum inhibitory concentration (MIC)

**Figure 2:**
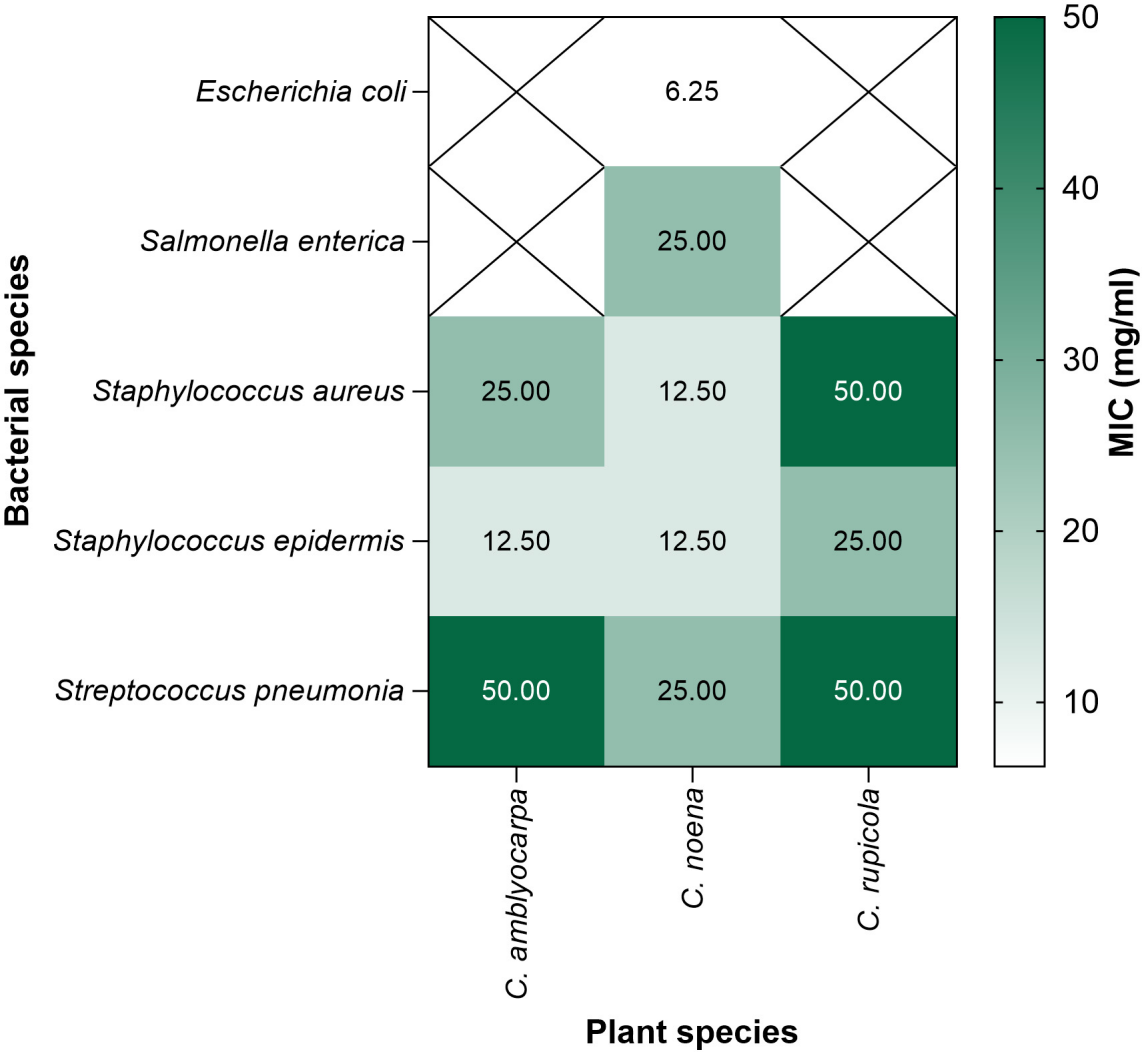
Minimum inhibitory concentration (MIC) values of *Cleome* extracts against bacterial isolates. Heatmap showing the mean MIC values (mg/mL, based on three replicates) for the ethanolic extracts of *C. amblyocarpa, C. noeana*, and *C. rupicola* against Gram-positive and Gram-negative bacteria. Lower MIC values indicate stronger antibacterial activity. Cells marked with X in the heatmap indicate no activity at the tested concentrations.

MIC data were statistically analyzed using non-parametric statistical analysis due to their discrete nature and the presence of tied values. Minimum inhibitory concentrations (MICs) differed across plant species and bacterial isolates (Figure 2). Kruskal–Wallis tests revealed significant differences among species for *Staphylococcus aureus, Staphylococcus epidermidis*, and *Streptococcus pneumoniae* (*H* = 8.0, *p* = 0.018 in each case). Pairwise Mann–Whitney tests did not remain significant after Holm adjustment, reflecting the small sample size and the presence of tied replicate values. Descriptively, the ethanolic extract of *C. noeana* consistently exhibited lower MICs than the extracts of other species. For *S. aureus*, MICs were 12.5 mg/mL for *C. noeana* extract, compared with 25 mg/mL for *C. amblyocarpa* extract and 50 mg/mL for *C. rupicola* extract. For *Staphylococcus epidermidis*, both *C. noeana* and *C. amblyocarpa* inhibited growth at 12.5 mg/mL, while *C. rupicola* extract required 25 mg/mL. For *S. pneumoniae, C. noeana* was inhibitory at 25 mg/mL, whereas C. amblyocarpa and C. rupicola were inhibited at 50 mg/mL. Against Gram-negative bacteria, only the ethanolic extract of *C. noeana* was active within the tested range, with MICs of 6.25 mg/mL for *E. coli* and 25 mg/mL for *S. enterica*.

#### 3.1.3 Minimum bactericidal concentrations (MBCs)

**Figure 3:**
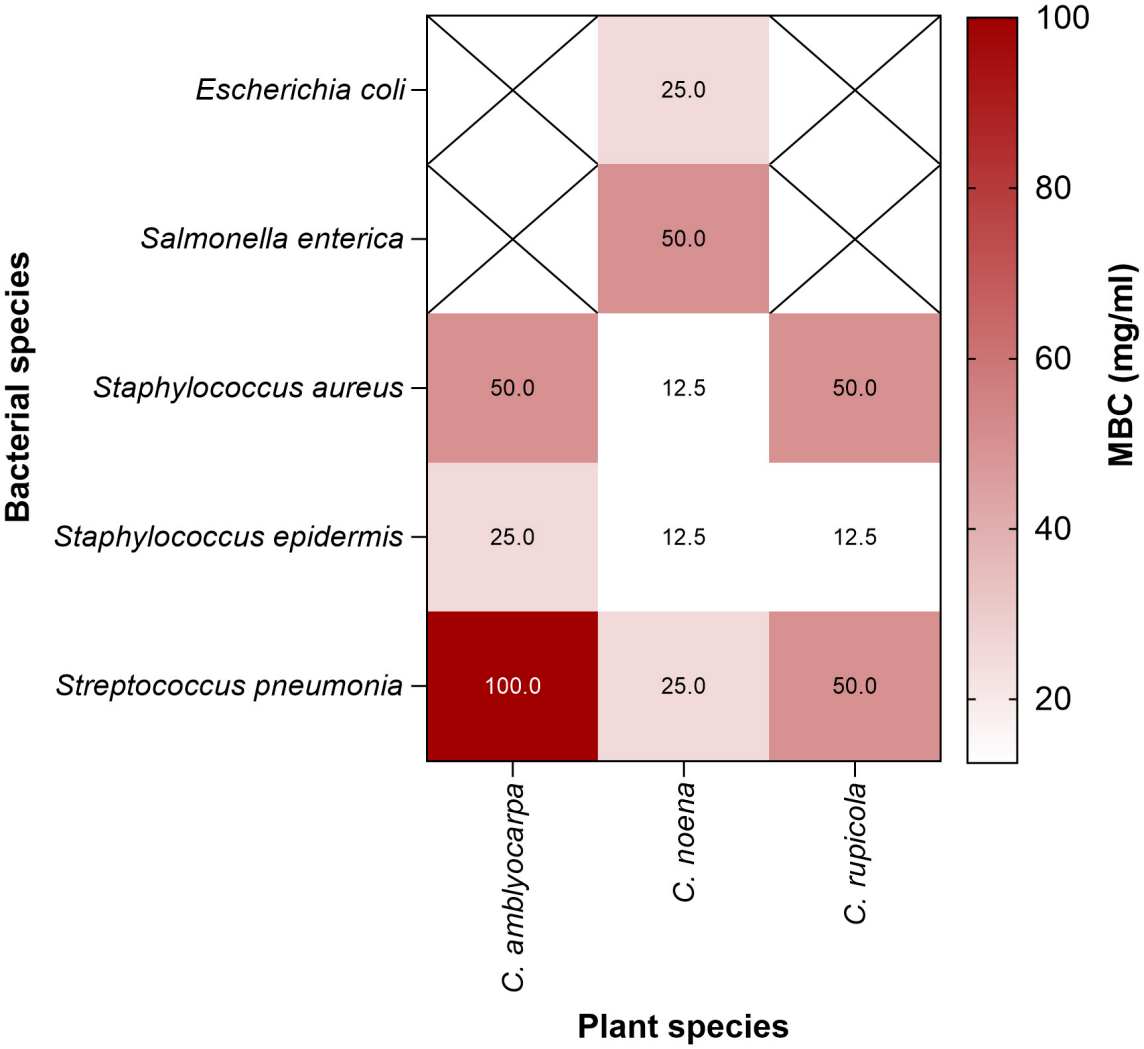
Minimum bactericidal concentrations (MBCs) of *Cleome* extracts. Heatmap showing MBC values (mg/mL) for the ethanolic extracts of *C. amblyocarpa, C. noeana*, and *C. rupicola* against Gram-positive and Gram-negative bacteria. Cells marked with X in the heatmap indicate combinations that were not determined.

MBC assays confirmed that inhibitory effects were often bactericidal (MBC/MIC ≤ 4). *C. noeana* extract was most potent: complete killing occurred at 12.5 mg/mL for *S. aureus* and *S. epidermidis*, 25 mg/mL for *E. coli* and *S. pneumoniae*, and 50 mg/mL for *S. enterica*. *C. rupicola* extract killed *S. epidermidis* at 12.5 mg/mL but required 50 mg/mL for *S. aureus* and *S. pneumoniae*. *C. amblyocarpa* extract was weakest, requiring 100 mg/mL for *S. pneumoniae*. Corresponding **MBC/MIC ratios were as follows:** *C. noeana*−1 for *S. aureus* and *S. epidermidis*, 2 for *E. coli* and *S. pneumoniae*, and 2 for *S. enterica*; *C. rupicola*−1 for *S. epidermidis* and 2 for *S. aureus* and *S. pneumoniae*; *C. amblyocarpa*−4 for *S. pneumoniae*. **All ratios were** **≤4, consistent with bactericidal activity** (Supplementary Table 1; Figure 3).

### 3.2 Correlations among diffusion assays, MIC, and MBC

**Figure 4:**
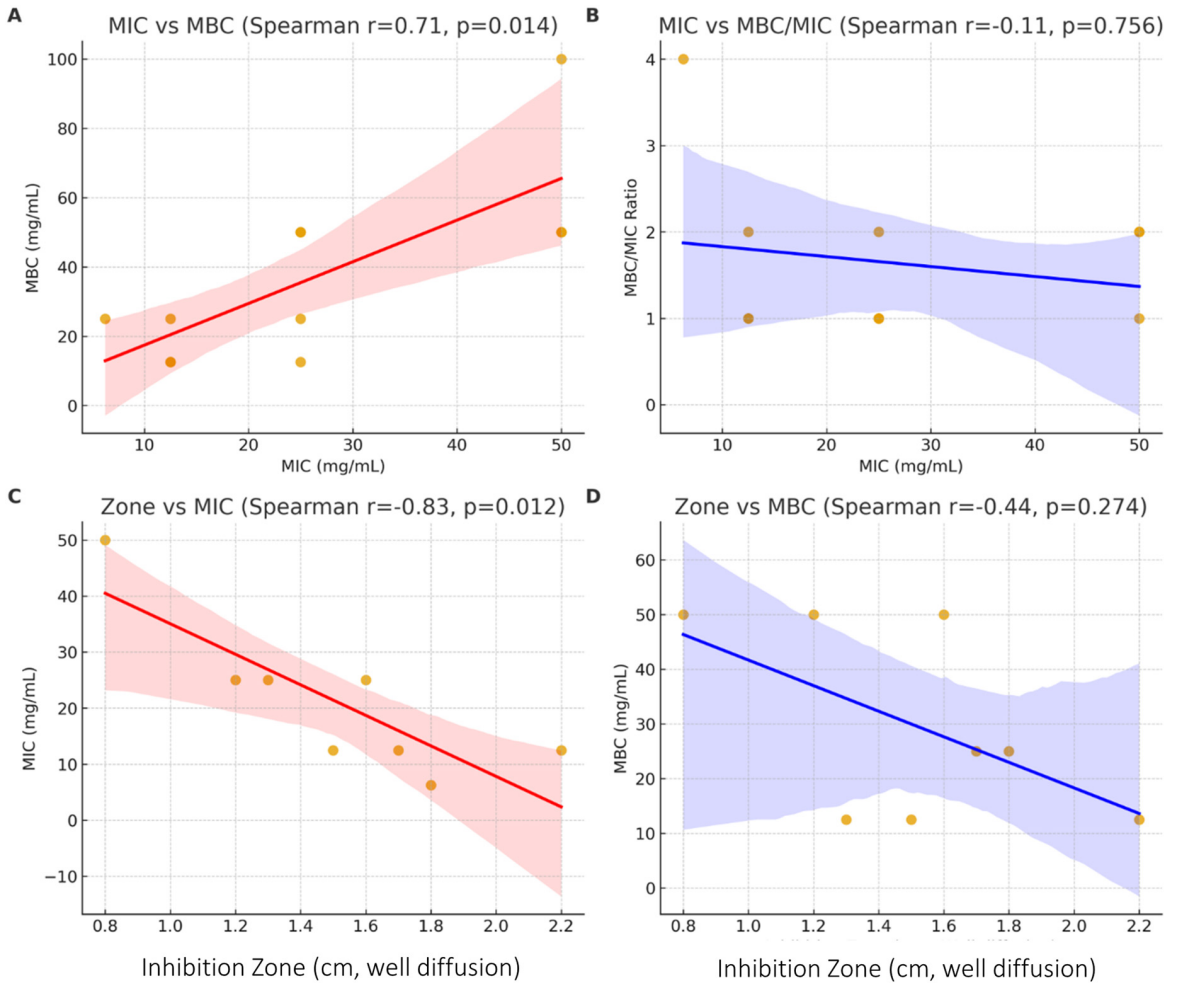
Correlation analyses between diffusion assay zones, MIC, and MBC values. **(A)** MIC vs. MBC. **(B)** MIC vs. MBC/MIC ratio. **(C)** Inhibition zones vs. MIC. **(D)** Inhibition zones vs. MBC.

The correlation analysis (Figure 4) revealed consistent relationships between agar diffusion results and broth-based assays. MIC values were positively correlated with MBC values (Figure 4A), indicating that isolates requiring higher concentrations for growth inhibition also needed higher concentrations to achieve bactericidal effects. By contrast, no clear relationship was observed between MIC and the MBC/MIC ratio (Figure 4B), suggesting that the bactericidal classification was independent of the absolute MIC value.

Inhibition zones obtained by the well diffusion method were strongly and inversely correlated with MIC values (Figure 4C), confirming that larger zones reflected lower inhibitory concentrations. However, the association between inhibition zones and MBC values was weaker and not statistically significant (Figure 4D), indicating that diffusion assays were more predictive of inhibitory than bactericidal thresholds.

In summary, the diffusion assay results aligned well with MIC values but were less consistent with MBC, highlighting the greater sensitivity of broth-based methods for identifying bactericidal activity. These correlations confirm the internal consistency of our antimicrobial assays and justify the focus on MIC as the most reliable comparative parameter.

### 3.3 Combined effects of *C. noeana* extract and bacteriophages

**Figure 5:**
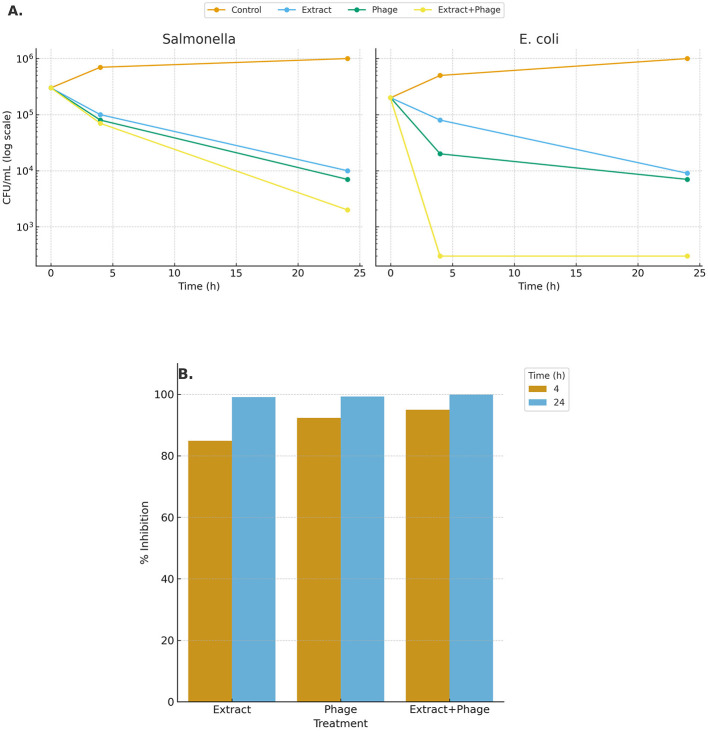
Effects of *Cleome noeana* extract, phage, and their combination on bacterial growth. **(A)** Bacterial counts (CFU/ml, log scale) of *Salmonella enterica* and *Escherichia coli* over 0–24 h under different treatments. **(B)** Percentage inhibition of bacterial growth at 4 h and 24 h relative to untreated controls calculated from the *E. coli* time–kill data in panel **(A)** only.

In time–kill assays against *E. coli*, untreated controls grew to ≈10^6^ CFU/mL by 24 h, whereas the combined *C. noeana* extract + phage treatment achieved a >3-log reduction (>99.9%) within 4 h and maintained near-zero CFU counts thereafter. Extract alone reduced *E. coli* counts by ~1 log (≈90%), and phage alone by ~2 logs (≈99%). The combination achieved a >3-log reduction (>99.9%), corresponding to a 1,000-fold decline compared to untreated controls. By 24 h, the combination maintained near-zero CFU counts, whereas the extract alone left detectable survival (Figure 5A, *E. coli* panel).

For *S. enterica*, suppression was slower than for *E. coli*, but the extract–phage combination still achieved clearance at least 2-fold faster than phage alone. Inhibition analysis confirmed that, while single treatments approached complete inhibition by 24 h, the combined therapy exceeded 99% inhibition within 4 h, demonstrating both greater potency and faster action (Figure 5A).

Percentage inhibition analysis based on the *E. coli* time–kill data further confirmed that while single treatments approached complete inhibition by 24 h, the combined therapy exceeded 99% inhibition within 4 h, demonstrating both greater potency and faster action (Figure 5B).

### 3.4 Antimicrobial metabolite profiles of *Cleome* species

**Figure 6:**
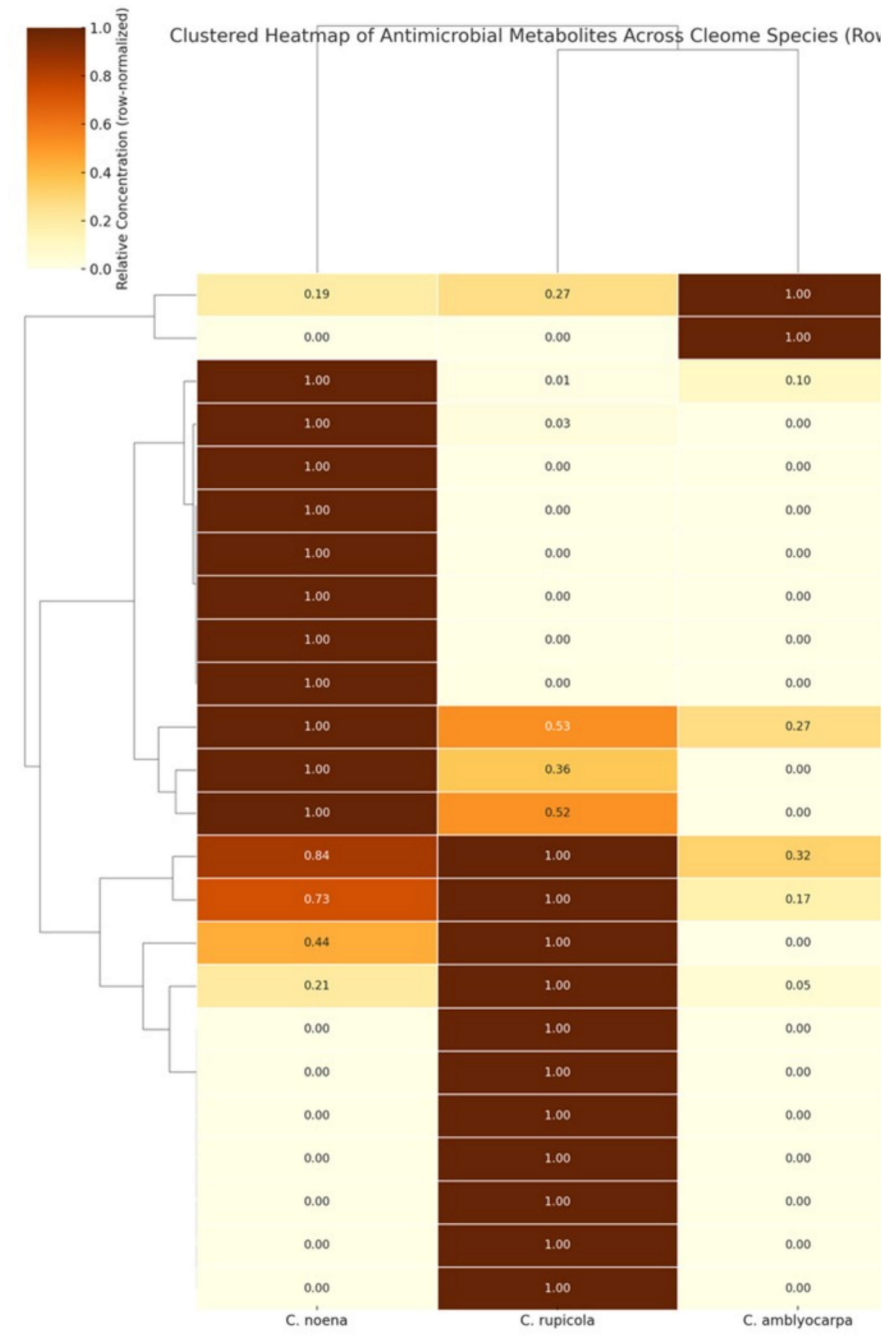
Clustered heatmap of metabolites in three *Cleome* species. Row-normalized concentrations (0–1 scale) of 25 metabolites (mean of *n* = 3 replicates) are shown for *C. rupicola, C. noeana*, and *C. amblyocarpa*. Hierarchical clustering highlights species-specific enrichment: hydroxycinnamates and unsaturated fatty acids in *C. rupicola*, caffeic acid in *C. noeana*, and sterols in *C. amblyocarpa*.

GC–MS analysis revealed overlapping yet distinct metabolite profiles among the three *Cleome* species (Supplementary Table 2, Figure 6). Palmitic, stearic, and caffeic acids were detected in all species but accumulated at markedly different abundances.

*Cleome noeana* was the most phenolic-rich, dominated by caffeic and oleic acids. Caffeic acid in *C. noeana* reached 7.48 × 10^8^ arbitrary units, ~10-fold higher than in *C. amblyocarpa* (7.19 × 10^7^) and ~92-fold higher than in *C. rupicola* (8.13 × 10^6^). Oleic acid was also enriched in *C. noeana*, ~2.8-fold higher than in *C. rupicola* (3.69 × 10^7^) and absent in *C. amblyocarpa*. Unique metabolites of *C. noeana* included linoleic acid ethyl ester and myristic acid. Notably, a compound matching 25-hydroxycholesterol was also detected exclusively in *C. noeana*. Because this oxysterol is classically described in animals and rarely validated in plants, its identification is considered tentative pending confirmation with authentic standards using techniques such as targeted LC–MS/MS or NMR spectroscopy (DeBarber et al., 2008).

*Cleome rupicola* was characterized by a hydroxycinnamate-rich profile, containing chlorogenic and coumaric acids, which were absent in the other two species. Stearic acid was highest in *C. rupicola* (4.29 × 10^8^), 1.4-fold greater than in *C. noeana* (3.13 × 10^8^) and ~6-fold greater than in *C. amblyocarpa* (7.20 × 10^7^). Palmitic acid also peaked in *C. rupicola* (6.37 × 10^8^).

*Cleome amblyocarpa* was enriched in sterols, particularly stigmasterol, which was ~3.7-fold higher than in *C. rupicola* (3.37 × 10^7^) and ~5.3-fold higher than in *C. noeana* (2.34 × 10^7^). Vanillylmandelic acid was uniquely present in *C. amblyocarpa*.

Thus, the three *Cleome* species exhibit distinct antimicrobial metabolite strategies: *C. amblyocarpa* shows a sterol-dominant profile, *C. rupicola* is hydroxycinnamate/saturated-fatty-acid rich, and *C. noeana* is phenolic/unsaturated-fatty-acid dominant.

### 3.5 Multivariate PLS–VIP analysis of metabolite–activity relationships

Partial Least Squares (PLS) regression with Variable Importance in Projection (VIP) scores (Table 2) identified the metabolites most strongly associated with antimicrobial activity. Metabolites with VIP > 1 included caffeic acid (VIP = 1.26), myristic acid, α-terpineol, and linoleic acid ethyl ester. These compounds were positively correlated with inhibition zones and negatively correlated with MIC values, confirming their contribution to stronger antibacterial effects.

**Table 2:** Top metabolites identified by PLS-VIP analysis and their correlations with antimicrobial activity indices.

Metabolite	VIP score	Spearman *R* inhibition zone	Spearman *R* MIC
Caffeic acid	1.2552	1.0	−1.0
Myristic acid	1.2194	0.866	−0.866
(S)-(–)-α-terpineol	1.2194	0.866	−0.866
Linoleic acid ethyl ester	1.2194	0.866	−0.866
β-sitosterol	1.218	0.5	−0.5
2,6-Dihydroxybenzoic acid	1.2175	0.5	−0.5
Campesterol	1.2067	0.5	−0.5
D-(-)-Lactic acid	1.0611	−0.866	0.866
11-Octadecenoic acid, (E)-	1.0611	−0.866	0.866

Sterols such as β-sitosterol and campesterol also ranked highly, suggesting possible roles in membrane disruption. In contrast, coumaric acid derivatives as well as D- and L-lactic acids showed weak or inverse associations with antimicrobial activity. These metabolites are enriched in *C. rupicola* (with D-lactic acid exceeding the VIP > 1 threshold and coumaric acids unique to this species), whereas *C. noeana* contained relatively higher levels of lactic acid itself. The dominance of these less-active compounds in *C. rupicola* is consistent with its reduced antibacterial potency. Taken together, these results highlight a small set of “signature metabolites” that likely underpin the superior activity of *C. noeana*.

### 3.6 Correlation of individual metabolites with antimicrobial activity

To complement the PLS–VIP analysis, pairwise Spearman correlations were computed between individual metabolite abundances and antimicrobial activity expressed as the mean inhibition zone (AvgZone). The results detailed in Supplementary Table 2, revealed a clear dichotomy between high- and low-activity compounds.

Several metabolites were strongly and positively correlated with inhibition zones (ρ > 0.85), including caffeic acid, (S)-(-)-α-terpineol, 25-hydroxycholesterol, linoleic acid ethyl ester, and myristic acid. These findings are consistent with their high VIP scores and confirm their roles as key antimicrobial metabolites. In contrast, chlorogenic acid, *m*-coumaric acid derivatives, and lactic acid esters exhibited negative correlations with inhibition zones (ρ < −0.80), reflecting their higher abundance in the less active species *C. rupicola* and *C. amblyocarpa*.

Together, this compound-specific analysis reinforces the multivariate results, highlighting that the distinct metabolic signature of *C. noeana* enriched in positively associated metabolites and depleted in negatively associated ones explains its superior antimicrobial activity (Supplementary Table 3).

## 4 Discussion

This study establishes the ethanolic extract of *Cleome noeana* as the most potent of three arid-region *Cleome* species tested. It consistently produced lower MICs and bactericidal ratios (MBC/MIC ≤ 4) compared to *C. rupicola* and *C. amblyocarpa*. Chemometric analyses linked its enhanced activity to enrichment in a small set of metabolites: caffeic acid, α-terpineol, myristic acid, and 25-hydroxycholesterol. In time–kill assays, the *C. noeana* extract was also compatible with bacteriophages, producing >3-log reductions within 4 h and accelerating clearance compared to single treatments. Together, these results nominate *C. noeana* as a lead desert-adapted species for antibacterial drug discovery.

### 4.1 Arid-region *Cleome* species demonstrate antibacterial potential

Our results extend previous reports of antibacterial activity in other *Cleome* congeners. *C. droserifolia*, a desert shrub from Egypt, has shown strong inhibition of *S. aureus, E. coli*, and *Salmonella* (Muhaidat et al., 2015; Hashem and Shehata, 2021). *C. spinosa* extracts inhibited both bacteria and fungi, with MICs < 1 mg/mL against *S. aureus*, and activity strongly correlated with phenolic content (Silva et al., 2016). By contrast, little is known about arid-region taxa such as *C. rupicola* and *C. amblyocarpa*. Our data show these species had limited or no effect against Gram-negative pathogens, confirming that antimicrobial potential is not uniform across the genus. Importantly, this is the first report of antibacterial activity in *C. noeana*, underscoring the need for comparative exploration across understudied desert species.

### 4.2 Signature metabolites explain potency of *C. noeana*

Metabolomic profiling highlighted caffeic acid, α-terpineol, myristic acid, and 25-hydroxycholesterol as key metabolites that drive antibacterial potency. *C. noeana* contained caffeic acid at levels ~9-fold higher than *C. amblyocarpa* and >90-fold higher than *C. rupicola*. Phenolic acids such as caffeic acid destabilize membranes, interact with DNA, and inhibit efflux pumps (Dos Santos et al., 2018; Sivakumar et al., 2020; Zakaria et al., 2017). α-Terpineol disrupts lipid bilayers and enhances membrane permeability (Huang et al., 2021; Yang et al., 2023; Li et al., 2014). Myristic acid inserts into bacterial membranes and may also alter DNA conformation (Chen et al., 2019).

A spectral match to 25-hydroxycholesterol was observed exclusively in *C. noeana*. Because this oxysterol is classically reported in animals and only rarely in plants, the assignment remains tentative pending confirmation with authentic standards. Importantly, the superior antibacterial activity of *C. noeana* can be explained by its enrichment in validated metabolites such as caffeic acid, α-terpineol, and myristic acid. Negative correlations with chlorogenic and coumaric acids help explain the weaker activity of *C. rupicola*. Collectively, *C. noeana's* superior activity reflects enrichment of high-impact metabolites with complementary mechanisms.

### 4.3 Plant–phage combinations accelerate bacterial clearance

Phage–antibiotic synergy is well documented (Gu Liu et al., 2020; Qin et al., 2024), but plant–phage interactions remain poorly studied. Reports range from synergistic suppression with *Alhagi maurorum* extract plus phage (Mirzaei et al., 2024) to antagonism, in which *Echinacea* extracts inhibited phage activity (Stachurska et al., 2023). Our study is the first to evaluate a *Cleome* extract in this context. The finding that *C. noeana* extracts accelerated phage-mediated killing (>99.9% inhibition at 4 h) is significant because it demonstrates compatibility rather than antagonism. The synergy likely arises from complementary actions: membrane-active phytochemicals lowering bacterial defenses while phages provide host-specific lysis. This dual-modality approach could slow resistance development and improve therapeutic outcomes.

### 4.4 Strengths and limitations

Strengths of this work include the integration of multiple antimicrobial assays, time–kill dynamics, and chemometric modeling. Limitations include reliance on crude extracts, absence of cytotoxicity testing, and lack of *in vivo* validation. Moreover, only three species were examined, limiting generalizability. Despite these caveats, the results provide a strong rationale for bioassay-guided isolation of metabolites, testing additive or synergistic interactions, and evaluating cytotoxicity and *in vivo* efficacy.

### 4.5 Implications for antimicrobial discovery from *Cleome*

These findings highlight *C. noeana* as a promising source of antibacterial metabolites. The identification of 25-hydroxycholesterol is particularly novel, hinting at convergent defensive chemistry across kingdoms. The demonstrated compatibility with bacteriophages positions *C. noeana* as a candidate for integrated therapies that combine phytochemicals with biologics. More broadly, this study emphasizes the untapped value of desert flora as reservoirs of specialized metabolites and supports systematic pharmacological evaluation of arid-region *Cleome* species.

## 5 Conclusion

This study provides the first report of antibacterial activity in arid-region *Cleome* species, identifying *C. noeana* as a particularly promising source of bioactive metabolites. Across all assays, *C. noeana* consistently outperformed *C. rupicola* and *C. amblyocarpa*, demonstrating bactericidal activity and enhanced clearance when combined with bacteriophages. Metabolomic profiling and chemometric analyses highlighted a small set of signature metabolites caffeic acid, α-terpineol, myristic acid, and 25-hydroxycholesterol that plausibly explain its potency.

These findings reinforce the value of desert-adapted flora as reservoirs of antimicrobial chemistry and underscore the translational potential of combining plant-derived metabolites with phages for accelerated bacterial suppression. Future investigations should prioritize bioassay-guided isolation of active compounds, mechanistic validation, and *in vivo* testing to confirm efficacy and safety. Collectively, the results nominate *C. noeana* as a lead candidate for natural product–based antibacterial discovery and as a phage-adjunct therapy in the context of rising antimicrobial resistance. Future work should include targeted supplementation and isolation of individual metabolites to directly validate their contribution to antibacterial activity.

## Data Availability

The original contributions presented in the study are included in the article/Supplementary material, further inquiries can be directed to the corresponding authors.
