# Supplementary material for: Integrated bioassays and metabolomics identify Cleome noeana as a promising antibacterial candidate
Source: Front Microbiol. 2026 Mar 27;17:1777863. doi: 10.3389/fmicb.2026.1777863 (PMC13066284; doi:10.3389/fmicb.2026.1777863)
Supplement: Supplementary file 1 [file Data_Sheet_1.docx]

**Supplementary Tables**

## **Supplementary Table S1**. Minimum inhibitory concentration (MIC), minimum bactericidal concentration (MBC), and MBC/MIC ratios of *Cleome* extracts against tested bacteria.

| Bacterial species | Plant species (extract) | MIC (mg/mL) | MBC (mg/mL) | MBC/MIC ratio | Interpretation |
| --- | --- | --- | --- | --- | --- |
| *Staphylococcus aureus* | *C. noeana* | 12.5 | 12.5 | 1 | Bactericidal |
| *Staphylococcus aureus* | *C. amblyocarpa* | 25 | 50 | 2 | Bactericidal |
| *Staphylococcus aureus* | *C. rupicola* | 50 | 50 | 1 | Bactericidal |
| *Staphylococcus epidermidis* | *C. noeana* | 12.5 | 12.5 | 1 | Bactericidal |
| *Staphylococcus epidermidis* | *C. amblyocarpa* | 12.5 | 12.5 | 1 | Bactericidal |
| *Staphylococcus epidermidis* | *C. rupicola* | 25 | 25 | 1 | Bactericidal |
| *Streptococcus pneumoniae* | *C. noeana* | 25 | 25 | 1 | Bactericidal |
| *Streptococcus pneumoniae* | *C. amblyocarpa* | 50 | 100 | 2 | Bactericidal |
| *Streptococcus pneumoniae* | *C. rupicola* | 50 | 50 | 1 | Bactericidal |
| *Escherichia coli* | *C. noeana* | 6.25 | 25 | 4 | Bactericidal |
| *Salmonella enterica* | *C. noeana* | 25 | 50 | 2 | Bactericidal |

Note: MIC and MBC values are expressed in mg/mL. The MBC/MIC ratio was calculated to assess bactericidal activity, where ratios ≤ 4 indicate bactericidal effects.

**Supplementary Table S2.** Average peak areas (arbitrary units) of 234 metabolites detected in three *Cleome* species (mean of *n* = 3 replicates). Values are derived from GC–MS analysis after derivatization. Metabolite identifications are based on spectral library matches (NIST/Wiley) and should be regarded as putative unless confirmed with authentic standards. Notably, a spectral match to 25-hydroxycholesterol was observed exclusively in *C. noeana*; because this oxysterol is classically described in animals and rarely validated in plants, this assignment remains tentative pending confirmation.

| Compound | *C. noeana* | *C. rupicola* | *C. amblyocarpa* |
| --- | --- | --- | --- |
| (S)-(-)-.alpha.-Terpineol | 2934661.444 | 0.0 | 0.0 |
| β-Sitosterol | 28195328.444 | 93362.444 | 0.0 |
| 11-Octadecenoic acid, (E)- | 0.0 | 125999246.333 | 0.0 |
| 2,6-Dihydroxybenzoic acid | 2522174.0 | 11171.0 | 0.0 |
| 25-Hydroxycholesterol | 133423779.433 | 0.0 | 0.0 |
| 4-Coumaric acid | 0.0 | 48665613.011 | 0.0 |
| 9-Octadecenoic acid, (E)- | 19393.444 | 261129658.767 | 0.0 |
| Caffeic acid | 748249675.543 | 8129378.332 | 71869947.433 |
| Campesterol | 11879561.889 | 331726.556 | 0.0 |
| Chlorogenic acid | 0.0 | 95408217.567 | 0.0 |
| D-(-)-Lactic acid | 0.0 | 42,157,668.444 | 0.0 |
| L-(+)-Lactic acid | 0.0 | 6993501.777 | 0.0 |
| Lactic Acid | 38154502.0 | 20149168.336 | 10465492.446 |
| Linoleic acid ethyl ester | 1528047.222 | 0.0 | 0.0 |
| m-Coumaric acid | 0.0 | 19159663.777 | 0.0 |
| Myristic acid | 4875951.667 | 0.0 | 0.0 |
| Oleic acid, (Z)- | 102445171.657 | 36928457.998 | 0.0 |
| Palmitic acid | 536650567.457 | 636603682.223 | 202192942.123 |
| Phytol, acetate | 463978.0 | 1046605.111 | 0.0 |
| Phytol | 38995357.89 | 20356100.334 | 0.0 |
| Stearic acid | 313304401.433 | 428894520.11 | 71956547.233 |
| Stigmasterol | 23414228.221 | 33742193.221 | 124430722.233 |
| Thymol-.beta.-d-glucopyranoside | 68336985.667 | 332261952.9 | 18068894.556 |
| Vanillylmandelic acid | 0.0 | 0.0 | 539784.222 |

**Supplementary Table S3.** Spearman correlation coefficients between metabolite concentrations and antimicrobial activity expressed as the mean inhibition zone (AvgZone). A positive correlation indicates greater metabolite abundance associated with larger inhibition zones, whereas negative correlation indicates less metabolite abundance.

| **Metabolite** | **Spearman R** |
| --- | --- |
| (S)-(-)-α-Terpineol | 0.866 |
| β-Sitosterol | 0.5 |
| 11-Octadecenoic acid, (E)- | -0.866 |
| 2,6-Dihydroxybenzoic acid | 0.5 |
| 25-Hydroxycholesterol | 0.866 |
| 4-Coumaric acid | -0.866 |
| 9-Octadecenoic acid, (E)- | -0.5 |
| Caffeic acid | 1.0 |
| Campesterol | 0.5 |
| Chlorogenic acid | -0.866 |
| D-(-)-Lactic acid | -0.866 |
| L-(+)-Lactic acid | -0.866 |
| Lactic acid | 0.5 |
| Linoleic acid ethyl ester | 0.866 |
| m-Coumaric acid | -0.866 |
| Myristic acid | 0.866 |
| Oleic acid, (Z)- | 0.5 |
| Palmitic acid | -0.5 |
| Phytol, acetate | -0.5 |
| Phytol | 0.5 |
| Stearic acid | -0.5 |
| Stigmasterol | -0.5 |
| Thymol-.beta.-d-glucopyranoside | -0.5 |
| Vanillylmandelic acid | 0.0 |
